# Changing transmission dynamics among migrant, indigenous and mining populations in a malaria hotspot in Northern Brazil: 2016 to 2020

**DOI:** 10.1186/s12936-022-04141-6

**Published:** 2022-04-19

**Authors:** Erica Anne Wetzler, Paola Marchesini, Leopoldo Villegas, Sara Canavati

**Affiliations:** 1 World Vision US, 34834 Weyerhaeuser Way South Federal Way, Washington, USA; 2 Department of Surveillance for Zoonotic and Vector Borne Diseases, Ministry of Health, Brasilia, Federal District, Brazil; 3 Association for Social and Civil Impact (ASOCIS), Tumeremo, Venezuela; 4 Global Development One, Silver Spring, MD USA

**Keywords:** Cross-border malaria, Imported malaria, Key populations, High risk groups, Migrant populations, Indigenous groups, Brazil, Mining malaria, Roraima

## Abstract

**Background:**

Roraima state is the northernmost state in Brazil and the primary border-crossing point between Brazil and Venezuela. The uncontrolled surge of malaria in Venezuela, coupled with mass migration of Venezuelans to neighbouring countries and the upward trend in informal mining in the state, pose a serious threat to the broader region, especially to migrant, indigenous and mining populations, jeopardizing malaria elimination efforts. This study describes changes in the epidemiological profile of malaria in Roraima state related to time, place and populations at risk from 2016 to 2020.

**Methods:**

De-identified malaria surveillance data were obtained from the Malaria Epidemiological Surveillance System from 2016 to 2020. Pearson’s chi-square tested differences between imported and autochthonous cases. Multivariable logistic regression was used to identify risk factors for imported versus autochthonous cases by demographic characteristics.

**Results:**

Odds of being an imported case were higher for *Plasmodium falciparum* cases (AOR = 2.08). However, as the number of cases from Venezuela decreased in 2020 following closure of the border, the proportion of *P. falciparum* cases increased markedly, from 6.24% in 2019 to 18.50% in 2020. Over the 5-year period, the odds of being an imported case among miners were about nine times higher than the general population (AOR = 8.99). The proportion of total malaria cases that were among indigenous people increased from 33.09% in 2016 to 54.83% in 2020. Indigenous children had a higher burden of malaria with over 40% of cases in children 0 to 9 years old, compared to 8% in non-indigenous children 0 to 9 years old. In some municipalities, place of infection differed from place of notification, with a large proportion of cases in these municipalities reporting in Boa Vista.

**Conclusions:**

Malaria remains a serious threat in Roraima state, especially among high-risk populations, such as miners, migrants, and indigenous people. As malaria cases have increased among indigenous people and miners, and the proportion of *P. falciparum* cases has increased, elimination efforts require understanding of these risk factors to tailor interventions appropriately. Furthermore, cross-border surveillance systems need to be urgently strengthened at formal and unofficial border points, especially since the border with Venezuela reopened in July 2021.

**Supplementary Information:**

The online version contains supplementary material available at 10.1186/s12936-022-04141-6.

## Background

According to the *World Malaria Report 2020*, malaria is a growing problem in the Americas with a population at risk of 139 million people [1]. The majority of malaria cases occur in the Amazon region. In 2019, four countries accounted for 91% of malaria cases in the Americas: Venezuela (53%), Brazil (20%), Colombia (13%) and Peru (5%). *Plasmodium vivax* is the most widespread of the malaria species (79.5%), followed by *Plasmodium falciparum* (20.5%), and mixed species (< 1%) [1]. Although there was significant progress towards malaria control and elimination in the Americas in the 2000s, continued progress has suffered in recent years due to the major increase in malaria in Venezuela—from 35,500 cases in 2000, rising to over 467,000 by 2019 [1, 2]. From 2016 to 2017, Venezuela had the largest rate of increase in reported malaria cases in the world [3].

In Brazil, 99% of malaria cases are concentrated in the Amazon region, which comprises nine states, including Roraima state. Roraima state lies in the northernmost part of the Amazon region, bordering Venezuela to the west and Guyana to the east. The region is characterized by high precipitation, humidity, and an extensive network of rivers [2, 4]. In 2019, approximately 89.3% of autochthonous malaria cases in Brazil were from *P. vivax* malaria. Only 10.7% (16,327) of cases were *P. falciparum* and mixed malaria [5].

Since the crisis in Venezuela began in 2015, more than 5.4 million Venezuelans have left their country [6]. An estimated 261,000 Venezuelans refugees and migrants are officially registered in Brazil [7]. Venezuelan migrants are often coming from malaria-endemic regions, transporting malaria with them as they cross borders. Cases in Venezuela are concentrated in Bolivar State, bordering Roraima state, where there has been a rise in cases since the political and economic crisis began [8]. The most widely used official border crossing between Venezuela and Brazil is in Pacaraima in northern Roraima state. From 2012 to 2018, Roraima was the largest receiving state in Brazil of cross-border malaria cases, with miners, males and people of working age more likely to be imported cases [9].

Though the influx of migrants dropped when the border between the two countries closed when the COVID-19 pandemic began in 2020, population movements shifted within Roraima state as more Brazilians sought work in the informal mining sector in municipalities in riverine forested areas located near indigenous populations. There was a sharp rise in illegal informal mining during 2019–2020 in some areas of the Amazon states of Brazil, including Roraima, especially in north western municipalities such as Alto Alegre and Mucajaí, which are also protected indigenous areas [10]. Malaria from these areas has been characterized as “gold mining-related malaria” [2], as mining activity creates a favourable environment for the reproduction of vector mosquitoes by dredging pools of water that serve as artificial breeding sites [11]. In addition, the heavy migration of mining workers between these areas promotes the movement of infected people, increasing the probability of transmission [12].

Another group at high risk of malaria is indigenous people, especially children, and some occupational groups, including miners, whom are particularly vulnerable to increased malaria transmission [13]. In Amazonas state of Brazil, *P. falciparum* malaria has been associated with indigenous populations [14], and in neighbouring Bolivar state in Venezuela, indigenous people have higher Annual Parasite Prevalence (API) than non-indigenous groups [15].

The uncontrolled surge of malaria centred in Bolívar state in Venezuela, coupled with mass migration of Venezuelans to and from neighbouring countries until 2018 and surges in informal mining in Roraima and other border states in Brazil, pose a serious threat to the broader region, especially to indigenous populations, jeopardizing efforts to achieve goals for disease control and elimination and facilitating drug resistance [8, 16–18]. Further, there is a risk that a regional malaria corridor will form from Bolívar to Roraima via the mass migration of displaced individuals who take advantage of the existing roads and trails connecting Venezuela and Brazil [19].

Previous studies of trends in malaria cases from Roraima state have analysed routine reporting of notifiable diseases through 2018 [9, 13, 20]. However, little is known about how the epidemiology of malaria in Brazil’s northernmost state has changed since 2018.

Hence, the objective of this study was to describe changes in the epidemiological profile of malaria in Roraima state in relation to time, place and populations at risk (migrants, indigenous and mining populations) from 2016 to 2020. The specific aims of this study are to (1) describe distribution of incident malaria cases in Roraima state by demographic characteristics, (2) compare trends and changes in cases of malaria over time, (3) describe distribution of incident malaria cases by indigenous people compared to non-indigenous people, (4) compare *P. vivax* and *P. falciparum* cases by demographic characteristics, (5) identify trends and risk factors for imported versus autochthonous cases, and (6) describe changes in malaria trends in municipalities in Roraima state where there have been increased mining activities.

## Methods

### Study site and population

**Fig. 1:**
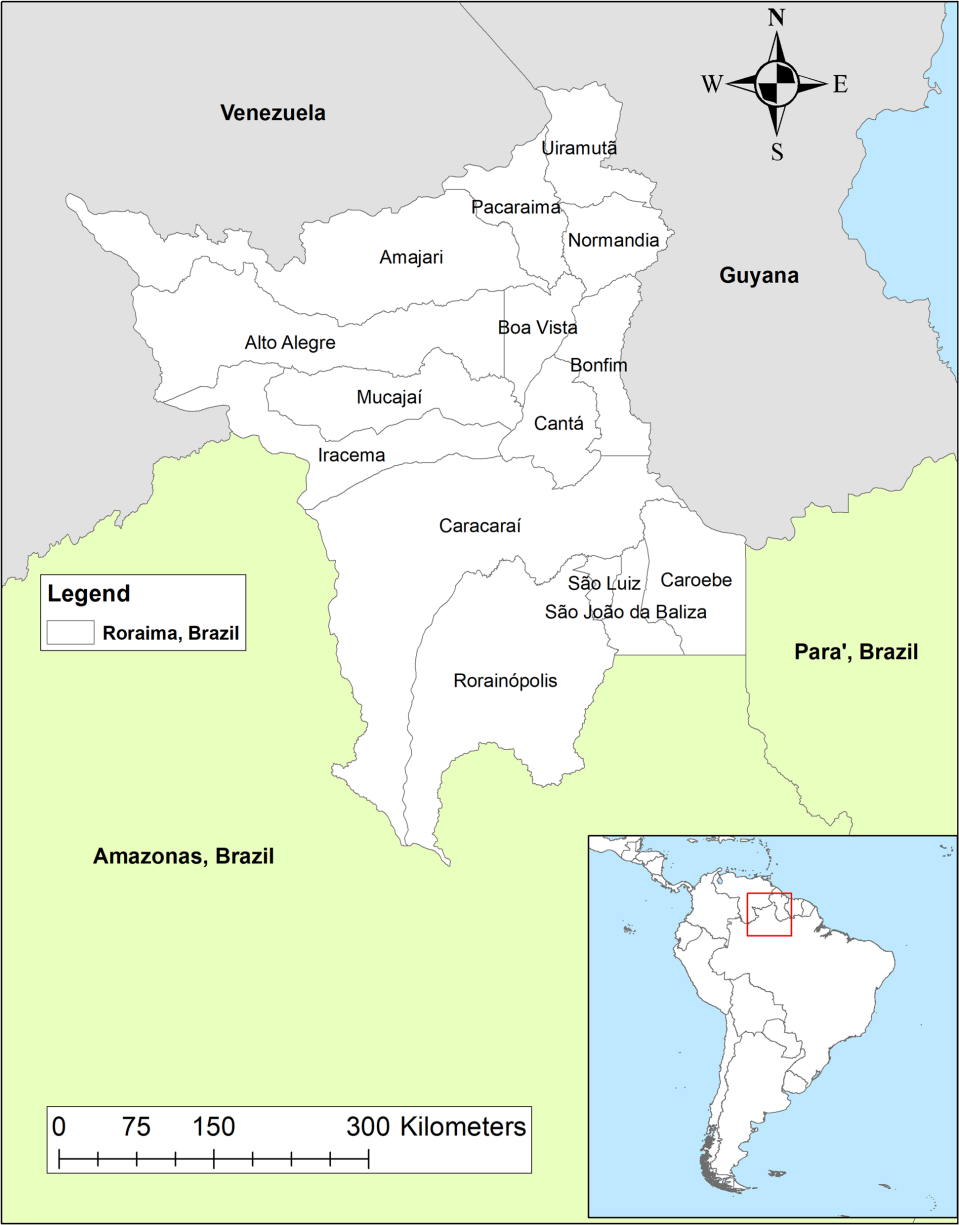
Municipal map of Roraima state, Brazil showing neighbouring states and countries

The Brazilian state of Roraima, in the northern part of the country, shares borders with Venezuela and Guyana. There are two internationally recognized border crossing points between Venezuela and Brazil at Santa Elena de Uairen in Bolívar state, Venezuela and Pacaraima in Brazil, and between Bonfim in Brazil and Lethem in Guyana (Fig. 1). There is one major paved road, Route 10, that runs along the eastern side of Bolívar and connects Venezuela with Pacaraima. This road crosses through Bolivar state's main endemic malaria region and in Brazil it connects Pacaraima with Boa Vista, Roraima’s largest city and the state capital. As of the 2020, the estimated population of Roraima was 631,181, with 66.5% of people residing in Boa Vista [21]. There are 15 municipalities in Roraima, five of which share a border with Guyana and five of which share a border with Venezuela (Fig. 1).

### Study design

#### Data sources

This is a descriptive study that utilizes malaria surveillance data from the Malaria Epidemiological Surveillance and Case Notification Information System (SIVEP-Malaria) in Brazil from 2016 to 2020.

In Brazil, malaria is a notifiable disease and must be reported. Since 2003, the National Malaria Prevention and Control Programme (PNCM) has used SIVEP-Malaria as the main tool for disease surveillance [22]. It is used for malaria case notification in the so-called endemic Amazonian area situated in northern Brazil and composed of nine states (Acre, Amazonas, Amapá, Roraima, Rondônia, Pará, Mato Grosso, Maranhão and Tocantins). Anti-malarials are available only through the National Health System (Sistema Único de Saúde (SUS)), cannot legally be purchased through private providers or pharmacies, and can only be dispensed with laboratory confirmed tests positive for malaria (slide, RDT or PCR). Hence, all diagnosed malaria cases in Brazil are notified through SIVEP.

The SIVEP surveillance system is hosted by the PNCM in the Ministry of Health’s Surveillance Secretariat, and documents data on all suspected or confirmed local and imported malaria cases collected via malaria surveillance reporting forms at any health facility in the country [20, 23]. SIVEP variables can also be filtered by place of notification (place of diagnosis) or place of probable infection. All malaria slides (positive and negative) should be notified to SIVEP; however, for this study, only positive slides were used.

### Case definitions

According to the guide on Health Surveillance in Brazil, the following are case definitions for malaria:

- Confirmed case: Clinical-laboratory criteria include all people in whom the presence of a malaria parasite or some component of the parasite has been identified in blood samples through laboratory test. SIVEP-malaria considers a positive slide as a malaria case.
- Excluded*:* Suspected case with a negative laboratory diagnosis for malaria. When there is strong epidemiological evidence of malaria, the test should be repeated within 24 to 48 h, or disease from another illness is confirmed.
- Incident malaria case: Occurrence of malaria illness/disease in a person in whom the presence of malaria parasites in the blood has been confirmed by parasitological testing [24]. In the SIVEP system, malaria cases occurring up to 60 days from previous treatment for *P. vivax* or 40 days for *P. falciparum* are counted as recurrences. There is no possible differentiation between relapse and recrudescence using routine diagnostic tools (slide and rapid diagnostic test, or RDT). After this period, cases are considered new cases. The slide that aims to ascertain if there has been a recurrence is called a “cure verification slide” (LVC in Portuguese). Slides for cure verification are not counted as a case, but as relapses.

### Study variables

Incident malaria cases were described by demographic, socio-economic, and malaria-related variables. These variables were categorized by calculating frequencies and percentages and identifying outliers and missing values. Additionally, when possible, categories were defined based on other studies. Details of the variables analysed are below.

Malaria cases per year (by notification date) by general demographic characteristics:

- Age group in years: 0–4 years old, 5–9 years old, 10–19 years old, 20–29 years old, 30–39 years old, 40–49 years old, and ≥ 50 years old.
- Sex: male and female
- Occupation: agriculture, domestic, tourism/travel, mining, timber/fishing, and other.
- Level of schooling: 1–4 years, 5–8 years, 9–11 years, and ≥ 12 years.
- Race: Indigenous groups and non-indigenous groups
- Country and state of infection: Roraima state, other Amazonian/endemic states in Brazil, Venezuela, Guyana and other countries.
- Municipality of infection (from Roraima state): Amajari, Alto Alegre, Boa Vista, Bonfim, Canta, Caracarai, Caroebe, Iracema, Mucajaí, Normandia, Pacaraima, Rorainópolis, São Joao, São Luiz, and Uiramutã.
- Municipality of notification (from Roraima state): Amajari, Alto Alegre, Boa Vista, Bonfim, Canta, Caracarai, Caroebe, Iracema, Mucajaí, Normandia, Pacaraima, Rorainópolis, São Joao, São Luiz, and Uiramutã

Imported vs. Autochthonous cases by*:*

- Species type: *P. falciparum* (includes mixed infection) and *P. vivax*
- Demographic characteristics
- Time from symptoms to treatment: number of days
- Year of notification: 2016, 2017, 2018, 2019, and 2020

Imported cases are defined as cases notified in Roraima state but with infection occurring outside of Roraima state, and further categorized as being imported from other Amazon states in Brazil, non-Amazon states in Brazil, or from other countries.

Indigenous people vs. non-indigenous people by:

- Species type: *P. falciparum* and *P. vivax*
- Demographic characteristics
- Time from symptoms to treatment: number of days
- Year of notification: 2016, 2017, 2018, 2019, and 2020

P. falciparum cases vs. P. vivax cases by:

- Cases in indigenous and non-indigenous people
- Demographic characteristics
- Time from symptoms to treatment: number of days
- Year of notification: 2016, 2017, 2018, 2019, and 2020

Annual Parasite Incidence (API):

The API was calculated as follows (20):

$$\frac{Confirmed\,cases\,or\,number\,of\,positive\,tests\,in\,the\,period}{{Total\,resident\,population\,in\,the\,period}} \times 1000\left( {{\text{inhab}}{.}} \right)$$

Shapefile files for maps were obtained from the Global Administrative Areas database (http://www.gadm.org/country). The maps were created using ArcMap 10.5.1 software (ESRI, Redlands, CA).

### Data analysis

The SIVEP database was used to analyse malaria surveillance data from Roraima state from 2016 to 2020. Analysis was performed using Stata® Version 14 (StataCorp LP, College Station, TX, USA). Cases were also sorted by *Plasmodium spp.* and by imported (infection in other states or other countries and notified in Roraima) and locally acquired cases. Differences in proportions of cases by imported versus autochthonous cases was assessed using Pearson’s chi-squared test of differences. Temporal trends were described from 2016 to 2020, by annual and monthly reported cases. A 60-day moving average was used to smooth the time series of malaria cases.

Multivariable logistic regression was used to identify risk factors for imported versus autochthonous cases by demographic characteristics. Case observations that had missing covariate data (e.g. race, age) were excluded from the final model, for a total of 82,268 cases in the adjusted model. For the education variable, a total of 22,344 cases were missing data for this covariate because it is not included for children; therefore, education was not included in the multivariate model. Crude odds ratios were generated for each possible risk factor by imported cases with autochthonous cases as the reference group, and those risk factors with corresponding p-values of < 0.05 were included in the final model. Crude odds ratios were compared with the adjusted odds ratios from the full regression model.

### Ethical considerations

This research uses secondary data, requested by the citizen information service of the Ministry of Health of Brazil. According to CNS Resolution 510/2016, research using publicly accessible information does not require the approval of the ethics committee. Pursuant to Law No. 12,527, of November 18, 2011, research using public domain information and research with databases can be used and aggregated if there is no possibility of individual identification and the research is carried out exclusively with scientific texts to review the scientific literature. Therefore, this study did not need to be submitted to National Research Ethics Commission (CONEP in Portuguese). All researchers involved declare no conflict of interest.

## Results

### Distribution of incident malaria cases in Roraima state by demographic characteristics

From 2016 to 2020, nearly two-thirds (61.98%) of cases were male, and this did not change notably between 2016 and 2020. The greatest share of cases was consistently in the 10 to 19 and 20 to 29-year-old groups, with 39.72% of cases among these two groups in 2016 and 42.82% in 2020 (Table 1). Of all notified cases, 10,136 (10.44%) were positive for *P. falciparum* and 85,519 (88.09%) for *P. vivax*.

**Table 1:** Proportional distribution of notified malaria cases by demographic characteristics in Roraima state, 2016–2020

	Characteristic	2016		2017		2018		2019		2020		Total	
		no. cases	percent	no. cases	percent	no. cases	percent	no. cases	percent	no. cases	percent	no. cases	percent
Age group (years)	0–4	743	9.13	1009	7.54	2029	8.87	2651	11.8	3581	13.04	10,013	10.61
	0–9	674	8.28	1007	7.52	2140	9.36	2428	10.81	3270	11.9	9519	10.09
	10–19	1439	17.68	2614	19.52	4491	19.64	4699	20.92	5866	21.35	19,109	20.26
	20–29	1796	22.07	2675	19.98	4744	20.74	4486	19.97	5628	20.49	19,329	20.49
	30–39	1596	19.61	2453	18.32	3884	16.98	3581	15.94	4057	14.77	15,571	16.51
	40–49	965	11.86	1818	13.58	2838	12.41	2437	10.85	2638	9.6	10,696	11.34
	50 +	926	11.38	1813	13.54	2746	12.01	2185	9.73	2432	8.85	10,102	10.71
Median age (years)			26.79		27.91		25.51		22.96		21.63		24.16
	Indigenous people		11.53		14.35		13.71		12.05		12.66		12.69
	Non-indigenous		30.78		30.32		30.04		30.57		31.14		30.57
	P. falciparum cases		32.58		34.65		32.06		28.43		23.43		28.17
	P. vivax cases		25.3		26.98		24.7		22.6		21.24		23.54
	Imported cases		31.8		32.12		30.76		29		29.63		30.8
	Autochthonous cases		20.21		25.27		23.09		21.83		21.27		22.21
Sex	Female	3520	39.25	5084	36.10	8497	36.36	8450	37.07	11,355	40.75	36,906	38.02
	Male	5449	60.75	8998	63.90	14,872	63.64	14,344	62.93	16,510	59.25	60,173	61.98
Race	Indigenous	2968	33.09	2952	20.96	7180	30.72	9761	42.82	15,277	54.83	38,138	39.29
	Non-indigenous	6001	66.91	11,130	79.04	16,189	69.28	13,033	57.18	12,588	45.17	58,941	60.71
Occupation	Agriculture	1214	13.99	3936	29.17	5453	26.65	4930	23.59	5503	22.22	21,036	23.82
	Domestic	361	4.16	623	4.62	1360	6.65	1477	7.07	3154	12.74	6975	7.9
	Tourism/travel	173	1.99	464	3.44	717	3.5	648	3.1	602	2.43	2604	2.95
	Mining	2833	32.64	2510	18.6	3993	19.51	3502	16.76	4567	18.44	17,405	19.71
	Timber/fishing	597	6.88	514	3.81	1050	5.13	3163	15.13	5310	21.44	10,634	12.04
	Other	3,501	40.34	5447	40.37	7892	38.56	7180	34.35	5627	22.72	29,647	33.57
Level of schooling (years)	None	1806	23.78	2416	19.37	3608	18.52	3131	19.19	4441	23.54	15,402	20.61
	1 to 4	1871	24.63	3141	25.19	4027	20.67	3155	19.33	3,669	19.45	15,863	21.23
	5 to 8	1837	24.19	3396	27.23	5919	30.38	4631	28.38	4,243	22.49	20,026	26.8
	9 to 11	1887	24.84	3088	24.76	5247	26.93	4886	29.94	5,907	31.31	21,015	28.12
	12 or more	195	2.57	429	3.44	683	3.51	517	3.17	605	3.21	2,429	3.25
Species	P. falciparum	1486	16.57	1204	8.55	2289	9.80	1423	6.24	5,155	18.50	11,557	11.90
	P. vivax	7483	83.43	12,878	91.45	21,080	90.20	21,371	93.76	22,710	81.50	85,522	88.10
Country of infection	Brazil	5715	63.72	11,170	79.32	18,265	78.16	20,049	87.96	26,774	96.08	81,973	84.44
	Venezuela	2470	27.54	2322	16.49	4478	19.16	2284	10.02	813	2.92	12,367	12.74
	Guyana	772	8.61	575	4.08	610	2.61	433	1.90	266	0.95	2,656	2.74
	Other	12	0.13	15	0.11	16	0.07	28	0.12	12	0.04	83	0.09
Imported		3306	36.86	3,027	21.50	5,213	22.31	2,845	12.48	1,201	4.31	15,592	16.06
Autohthonous		5663	63.14	11,055	78.50	18,156	77.69	19,949	87.52	26,664	95.69	81,487	83.94
Time from symptoms to treatment	Less than 24 h	2447	27.68	2,410	17.62	4,650	20.36	832	22.67	6,441	25.56	20,910	22.62
	24 to 48 h	1441	16.3	2,233	16.33	3,692	16.16	55	19.48	4,785	18.99	16,416	17.76
	More than 48 h	4952	56.02	9,034	66.05	14,502	63.48	12,662	57.85	13,978	55.46	55,128	59.63
Municipality of notification	Amajari	919	10.25	441	3.13	1,148	4.91	2,069	9.08	3,716	13.34	8,293	8.54
	Alto Alegre	1225	13.66	823	5.84	1,687	7.22	3,899	17.11	6,896	24.75	14,530	14.97
	Boa Vista	2369	26.41	3,671	26.07	5,713	24.45	6,465	28.36	6,531	23.44	24,749	25.49
	Bonfim	42	0.47	45	0.32	136	0.58	138	0.61	426	1.53	787	0.81
	Canta	134	1.49	1,628	11.56	2,155	9.22	1,049	4.6	841	3.02	5,807	5.98
	Carcarai	376	4.19	1,627	11.55	2,164	9.26	845	3.71	706	2.53	5,718	5.89
	Caroebe	249	2.78	127	0.9	264	1.13	686	3.01	592	2.12	1,918	1.98
	Iracema	145	1.62	602	4.27	763	3.27	1,069	4.69	1,308	4.69	3,887	4.00
	Mucajai	619	6.9	747	5.3	716	3.06	933	4.09	1,141	4.09	4,156	4.28
	Normandia	5	0.06	4	0.03	8	0.03	80	0.35	24	0.09	121	0.12
	Pacaraima	1444	16.1	1,250	8.88	3,674	15.72	1,857	8.15	2042	7.33	10,267	10.58
	Rorainopolis	831	9.27	2,955	20.98	3,603	15.42	1,981	8.69	1741	6.25	11,111	11.45
	Sao Joao	488	5.44	91	0.65	475	2.03	634	2.78	638	2.29	2326	2.40
	Sao Luiz	50	0.56	46	0.33	199	0.85	142	0.62	94	0.34	531	0.55
	Uiramuta	73	0.81	25	0.18	664	2.84	947	4.15	1169	4.2	2878	2.96

### Trends and changes in cases of malaria over time

**Fig. 2:**
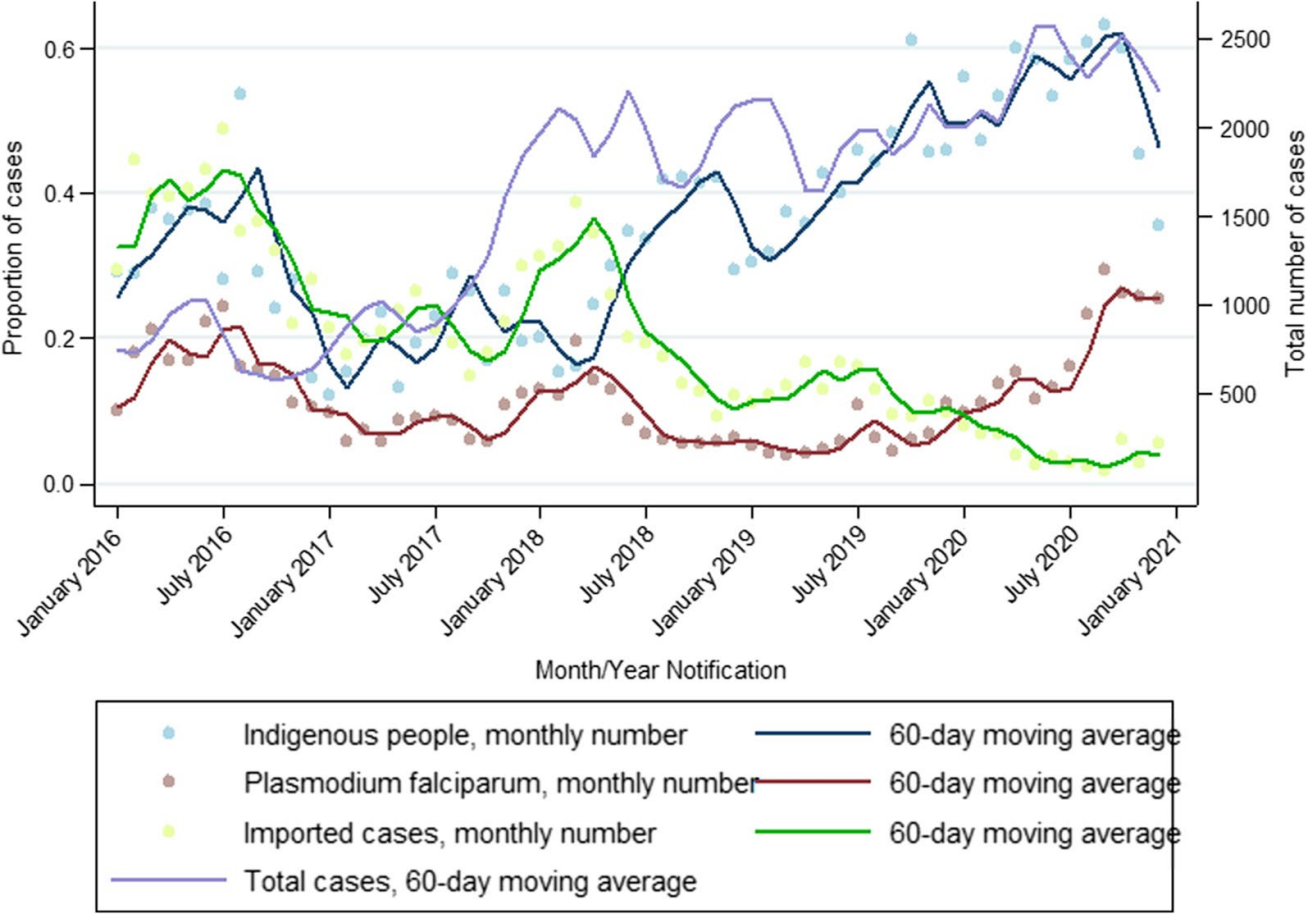
Total reported malaria cases per month in Roraima state, and the proportion of cases imported into the state, the proportion of *P. falciparum* cases, and the proportion of cases among indigenous groups, 2016–2020

From 2016–2020, the trend shows an increasing number of malaria cases in Roraima, with 8,969 cases in 2016, increasing steadily to 27,865 in 2020 for a total of 97,079 malaria cases over the 5-year period (Table 1). From January 2017, on average, less than 10% of monthly cases were *P. falciparum*, and this remained relatively stable until early 2020. By the last quarter of 2020, nearly 30% of all reported cases were *P. falciparum* (Fig. 2). The proportion of cases that were imported was highest in 2016 at over 40% in July 2016, and began to decrease steadily in March 2017, and by July 2020, less than 18% of cases were imported (Fig. 2).

The demographic characteristics with the greatest changes in proportion of malaria cases over time were race (categorized as indigenous and non-indigenous populations), occupation, and country of infection. Race was categorized as those cases who self-identified as indigenous versus non-indigenous, with the lowest proportion of indigenous cases in 2017 (20.96%), increasing steadily starting in March 2018 to a majority of total cases in 2020—by July 2020, over 60% of cases were reported as being in indigenous people (Fig. 2). The proportion of cases in the younger age groups, from 0 to 9 years, rose over time, with younger children accounting for nearly a quarter of malaria cases in 2020 (24.93%) compared to 17.41% in 2016 (Table 1).

### *Plasmodium vivax* and *P. falciparum* cases for different demographic characteristics

**Fig. 3:**
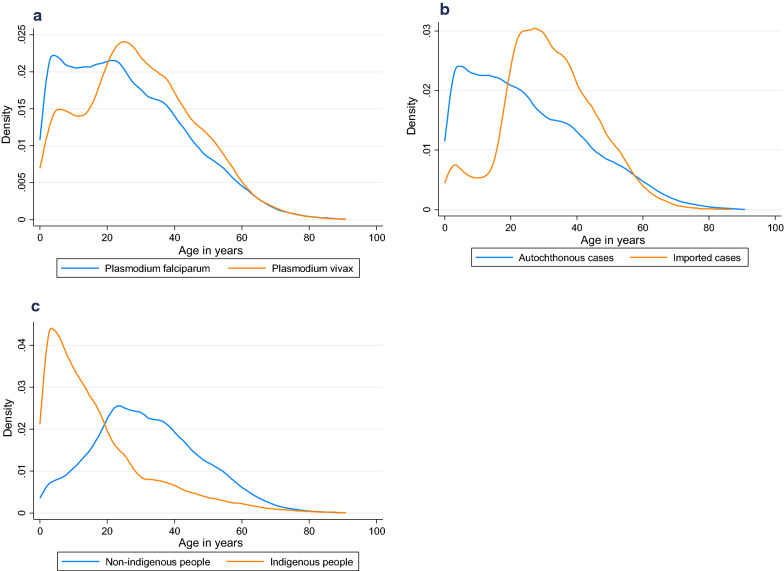
Proportion of malaria cases at each age, from 2016–2020. Density plots of age distribution of cases by case type: **a** *P. falciparum* and *P. vivax* cases, **b** autochthonous and imported cases, **c** cases in indigenous and non-indigenous people

Overall *P. falciparum* cases were younger than *P. vivax* cases (Fig. 3a). Among all cases, the median age of *P. falciparum* cases decreased every year from 32.58 years in 2016 to 23.43 years in 2020, while median age of *P. vivax* cases also decreased from 25.30 years in 2016 to 21.24 years in 2020. From 2019 to 2020, the number of cases of *P. falciparum* malaria increased in all age groups, with the largest increases in the 0–4 and 5–9 age groups (Table 1).

**Fig. 4:**
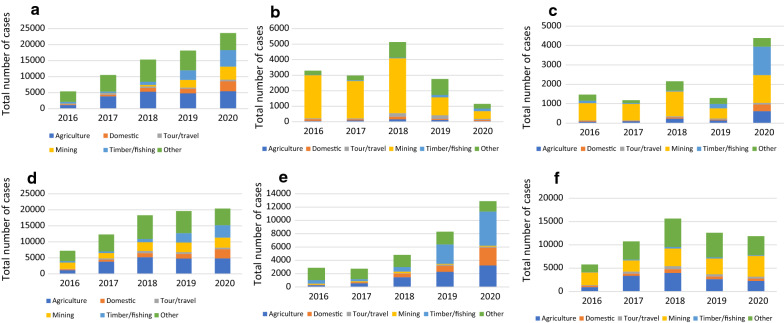
Occupations of reported malaria cases by year in Roraima state, 2016–2020, by reported occupation of the notified case by: **a**, **b** autochthonous and imported cases, **c**, **d** *P. falciparum* and *P. vivax* cases, and, **e**, **f** indigenous and non-indigenous people

*Plasmodium falciparum* cases reported working predominantly in mining (47.3% overall) compared to only 15.99% of *P. vivax* cases; the majority of these cases were in the “other” occupation category (35.93%) (Fig. 4c, d).

### Distribution of incident malaria cases by indigenous people compared to non-indigenous people

Malaria cases among indigenous people tended to be younger, with the median age at 12.69 years compared to non-indigenous people at 30.57 years (Table 1, Fig. 3c). Indigenous children had a higher burden of malaria with over 40% of cases in children 0 to 9 years, compared to non-indigenous children who had only 8% of cases among children 0 to 9 years (Fig. 3c and Additional file 1: Table S1). During the study period, there were differences in occupations of cases in indigenous versus non-indigenous people. The share of cases in indigenous people reporting timber or fishing as their main occupation increased over time, becoming the primary occupation by 2020. Cases in non-indigenous people were predominantly in the mining sector or reported as “other,” with those reporting mining increasing from 2016 to 2020 (Fig. 4e, f).

Over the five-year period, about 40.38% of cases received treatment within 48 h of symptoms. Time from symptoms to treatment differed by race: 44.01% of cases in indigenous people reported receiving treatment within 24 h compared to only 9.77% of non-indigenous people (Table 1).

### Trends and risk factors for imported versus autochthonous cases

In 2016, about two-thirds of notified infections reported the probable infection place as in Brazil (63.72%), compared to 96.08% in 2020 (Table 1). The number of imported cases peaked in 2018, with 4,478 cases originating in Venezuela (place of diagnosis in Roraima and reported place of infection as Venezuela) and 610 cases from Guyana. From 2016 to 2020, about 27.08% of imported cases were *P. falciparum*, compared to 9.00% of autochthonous cases (p-value < 0.001). Autochthonous cases tended to be younger than imported cases (Fig. 3b). Further, over time, there was also an increase in locally acquired malaria among miners, especially among locally acquired cases in Roraima. Mining only accounted for 61 locally acquired cases in 2016 compared to 4073 in 2020 (Fig. 4a).

Associations between imported versus autochthonous malaria cases and possible risk factors were all strong. The results of the multivariable logistic regression analysis to identify risk factors for imported malaria, with the respective adjusted OR values are presented in Table 2. The final model included age group, race, occupation, species of malaria, and time to treatment in days, and excluded observations with missing values.

**Table 2:** Multivariable logistic regression analysis of risk factors for imported versus autochthonous malaria cases, Roraima state, 2016–2020

Characteristic		Imported Cases		Autochthonous Cases		chi-square p-value	Adjusted OR		
		no. cases	percent	no. cases	percent		AOR	95% CI	p-value
Age group (years)	0–4	633	4.13	9,380	11.87	< 0.0001	1.00		
	5–9	427	2.79	9,092	11.51		0.70	0.61, 0.80	< 0.001
	10–19	1,600	10.45	17,509	22.16		0.81	0.73, 0.91	< 0.001
	20–29	4,613	30.12	14,716	18.62		1.11	1.00, 1.24	0.05
	30–39	3,946	25.77	11,625	14.71		1.13	1.01, 1.26	0.03
	40–49	2,518	16.44	8,178	10.35		1.09	0.96, 1.21	0.16
	50 +	1,576	10.29	8,526	10.79		0.88	0.78, 0.99	0.04
	Median age		30.8		22.21				
Sex	Female	5,408	34.68	31,498	38.65	< 0.0001	1.00		
	Male	10,184	65.32	49,989	61.34		0.88	0.84, .93	< 0.001
Race	Non-indigenous	1,488	9.54	36,650	44.98		1.00		
	Indigenous	14,104	90.46	44,837	55.02		0.38	0.35, 0.41	< 0.001
Occupation	Other	2,882	18.83	26,765	36.67	< 0.0001	1.00		
	Domestic	500	3.27	6,475	8.87		0.93	0.84, 1.04	0.20
	Tourism/travel	648	4.23	1,956	2.68		2.53	2.28, 2.79	< 0.001
	Mining	10,357	67.68	7,048	9.66		8.99	8.52, 9.48	< 0.001
	Timber/fishing	442	2.39	10,192	13.96		0.66	0.58, 0.75	< 0.001
	Agriculture	475	3.1	20,561	28.17		0.21	0.19, 0.24	< 0.001
Level of schooling (years)	None	806	5.7	14,596	24.09	< 0.0001	–	–	
	1 to 4	2,516	17.8	13,347	22.03		–	–	
	5 to 8	4,883	34.54	15,143	24.99		–	–	
	9 to 11	5,137	36.34	15,878	26.2		–	–	
	12 or more	795	5.62	1,634	2.7		–	–	
Species	*P. vivax*	4,223	27.08	7,334	9.00	< 0.0001	1.00		
	*P. falciparum*	11,369	72.92	74,153	91.00		2.08	1.96, 2.20	< 0.001
Time from symptoms to treatment	Less than 24 h	2,001	13.06	18,909	24.51	< 0.0001	1.00		
	24 to 48 h	1,958	12.78	14,458	18.74		0.80	0.74, 0.87	< 0.001
	More than 48 h	11,362	74.16	43,766	56.74		0.94	0.88, 1.01	0.09

The multivariate logistic regression model showed that cases among miners were 8.99 times more likely to be imported cases than the reference group (AOR = 8.99, 95% CI 8.52, 9.48), while reported occupation as timber/fishing and agriculture were 34% and 79% less likely to be an imported case (AOR = 0.66, 95% CI 0.58, 0.745 and AOR = 0.21, 95% CI 0.19, 0.24 respectively) (Table 2). Indigenous people had 62% lower odds of being an imported case than cases in non-indigenous people (AOR = 0.38, 95% CI 0.35, 0.41*). Plasmodium falciparum* cases were about two times more likely to be imported than *P. vivax* cases. (AOR = 2.08, 95% CI 1.96, 2.20). Cases treated between 24 and 48 h from symptoms had 20% lower odds of being imported compared to local cases (AOR = 0.80, 95% CI 0.74, 0.87). The odds of a case being imported were 30% less and 19% less in those aged 5 to 9 and 10 to19 compared to 0- to 4-year-olds (AOR = 0.70, 95% CI 0.61, 0.80; AOR = 0.81, 95% CI 0.73, 0.91), respectively.

### Changes in malaria trends in municipalities in Roraima state where there have been increased mining activities

Distribution of malaria cases by municipality of infection revealed that some municipalities such as Pacaraima and Rorainopolis saw a marked rise in notified cases between 2016 and 2018, aligning to the time period when cases with reported place of infection in Venezuela increased, followed by reductions in caseloads in 2019 and 2020. Notably in Alto Alegre, the total number of notified cases increased from 1,225 in 2016 to 6,896 in 2020 (Table 1). Boa Vista also showed an increased number of notified cases from 2016 to 2020.

Alto Alegre’s estimated population in 2020 was 15,380, so the API in 2020 was 740.31 cases/1000, about 10 times higher than the API for Roraima state (42.24 cases/1000) (Table 3). The districts with the greatest increase in API between 2016 and 2020 are Amajari, Alto Alegre, Iracema, Bonfim, and Uiramutã. The first three municipalities are in the north western part of the state, where there has been a reported uptick in illegal mining activity. Bonfim and Uiramutã municipalities are on the border with Guyana.

**Table 3:** Annual Parasite Index (API) by municipality of infection, Roraima state, Brazil, 2016 to 2020

Municipality	2016	2017	2018	2019	2020	Total
Alto Alegre	109.88	70.24	164.68	450.06	740.31	307.03
Amajari	66.53	34.08	92.34	137.91	268.08	119.79
Boa Vista	0.07	0.41	0.54	0.39	0.75	0.43
Bonfim	3.29	5.36	11.75	11.12	41.01	14.51
Canta	12.96	144.93	178.14	89.12	69.90	99.01
Caracarai	19.28	88.67	119.74	47.52	34.91	62.03
Caroebe	27.54	12.43	26.83	77.10	65.68	41.92
Iracema	19.26	80.21	76.21	98.83	117.44	78.39
Mucajai	38.87	48.07	54.83	91.75	118.53	70.41
Normandia	0.48	0.66	1.72	6.91	3.21	2.60
Pacaraima	14.99	24.97	101.67	99.30	103.16	68.82
Rorainopolis	27.85	104.31	120.51	65.15	55.26	74.61
Sao Joao da Baliza	62.92	8.14	49.80	66.33	59.18	49.27
Sao Luiz	8.80	9.35	28.24	21.54	20.72	17.73
Uiramuta	7.35	1.83	64.02	93.95	113.73	56.17
TOTAL	11.01	21.15	31.49	32.93	42.24	27.77

**Fig. 5:**
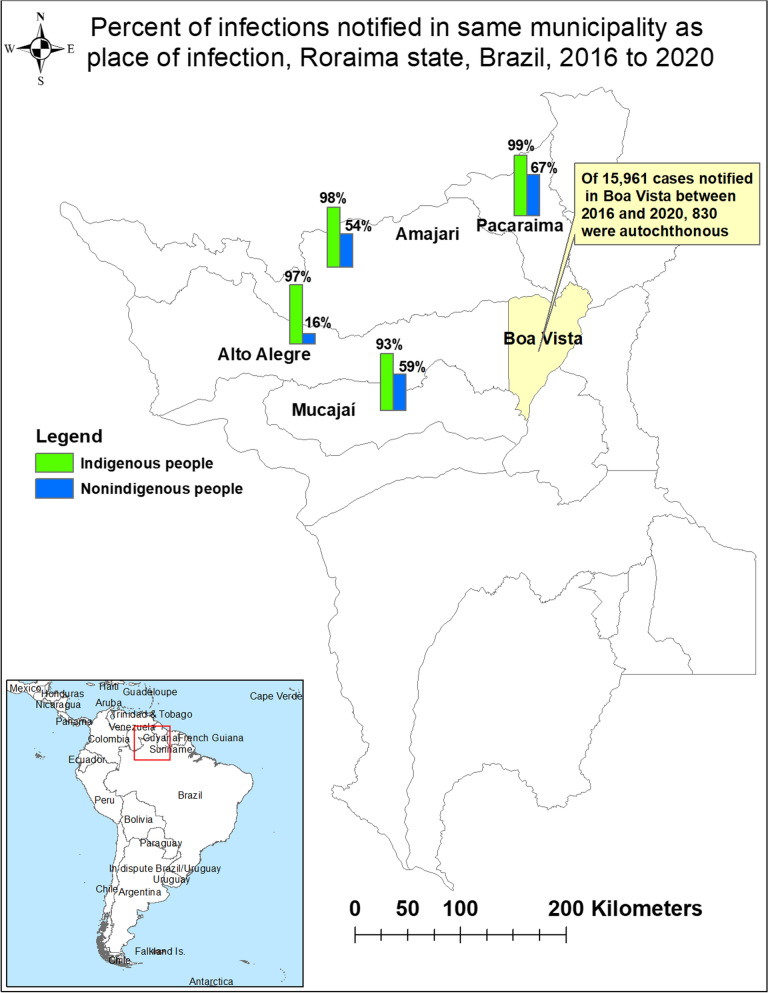
Differences in place of notification and place of infection for key municipalities in Roraima state, Brazil, 2016 to 2020

By municipality of notification, Alto Alegre municipality experienced the greatest surge in absolute number of notified cases from 2016 to 2020 (Table 1). However, analysis of municipality of infection against municipality of notification shows that for some municipalities, place of infection differed from place of notification, especially among non-indigenous people. In Alto Alegre and Mucajaí, both of which are areas with elevated illegal mining activity, there was a large proportion of infections notified in Boa Vista, while in Boa Vista and Pacaraima, there are few infections acquired locally compared to notifications (Fig. 5). In Boa Vista, there were only 830 local infections from 2016 to 2020, compared to 15,961 notifications. For infections coming from outside Brazil, those from Guyana were more often notified in Boa Vista, while those cases from Venezuela were notified in Pacaraima or Boa Vista.

## Discussion

During the 5-year study period, there were four notable shifts in the epidemiology of malaria in Roraima state, described in detail below.

### Increased imported malaria cases from Venezuela and Guyana

From 2016 to 2018, there was an increase in imported malaria cases from Venezuela and Guyana. The number of imported cases peaked in 2018, with 4,478 cases reported as originating in Venezuela and 610 cases from Guyana. However, this trend reversed in 2019 and even more so in 2020, when the border to Venezuela closed to mitigate spread of COVID-19. Similar to another study in Roraima from 2016 to 2018, *P. falciparum* cases had double the odds of being imported [9]. These findings demonstrate that humanitarian events within and outside Brazil, such as the crisis in Venezuela and the global COVID-19 pandemic can shift migration patterns and livelihoods activities quickly, in turn shifting the epidemiology of malaria. Resources and systems are needed so that control efforts can respond nimbly to rapid epidemiological shifts and changes in transmission dynamics. Since the border reopened with Venezuela in July 2021, there has been an influx of Venezuelan migrants again through Pacaraima municipality, and there is evidence that there will be an increase in malaria cases, especially *P. falciparum* [25]. Hence, innovative active surveillance approaches are needed to identify asymptomatic infections and artemisinin-resistant infections [26, 27].

### Shifts in *Plasmodium* species

Next, there were shifts in *Plasmodium* spp. The share of *P. falciparum* cases increased over time, peaking in 2020 at 18.50%, and was strongly associated with imported cases originating from Venezuela and Guyana. Over the 5-year period, 27.08% of imported cases were *P. falciparum*, compared to 9.00% of autochthonous cases. There were increases in the proportions of *P. falciparum* among indigenous people, especially in 2020. Thus, during the initial years of the analysis (2016–2017), the majority of *P. falciparum* malaria cases came from outside Brazil, primarily from Venezuela, where access to diagnostics and treatment was low. However, it is possible that at that time, the diagnostic network and health system in Roraima could support the increased burden from Venezuela, avoiding epidemics (at the time, there was increased national financial and human resources support to Roraima and increased state-level support to Pacaraima and other municipalities). By 2019 and 2020, the increase in *P. falciparum* cases occurred in indigenous areas, where gold mining was taking place. In the region, both indigenous reserves and illegal gold mining areas are characterized by lack of access to malaria diagnostics and treatment, allowing *P. falciparum* malaria to spread more widely. By species, whilst *P. falciparum* cases reported working predominantly in mining (47.3% overall), only 15.99% of *P. vivax* cases said they were miners. In the Guyana shield, which includes Roraima, mining is a known risk factor for malaria, and especially for *P. falciparum* malaria [28]. Further, there is evidence of an association between *P. falciparum* malaria and gold mining observed in neighbouring French Guiana [28].

### Strong links between mining and imported malaria

The analysis showed a strong link between mining and imported malaria, with miners nearly nine times more likely to be imported cases. This is aligned with other evidence from Roraima from 2016 to 2018 showing that mining was a strong correlate with cross-border malaria [9]. Over the study five-year period, the primary occupation of imported cases was in mining, but the proportion of autochthonous cases reporting their primary occupation as mining increased nine-fold from 2018 to 2020. This is compatible with findings in other mining regions of Brazil, such as Tapajós, where transmission in mining areas increased 17.8% in the first part of 2020 [29]. In Guyana, 94% of reported malaria cases occurred in major gold mining regions, while in Venezuela, gold mining accounted for ~ 60% of countrywide cases, demonstrating the potential for small, isolated, malaria-dense populations where there are minimal resources to stop outbreaks, reversing progress toward elimination [8, 28]. Evidence from Venezuela has shown that these illegal mining areas not only sustain transmission, but also can restore it after interventions have reduced malaria locally or even achieved local elimination in other areas, putting these areas at the crux of elimination efforts [8, 30].

### Increased malaria cases in indigenous populations over time

Finally, there was a surge in malaria cases indigenous people, linked with elevated illegal mining activities in protected indigenous areas. Most informal and illegal mining operations in Roraima are located on indigenous Yanomami reserves. Between 2017 and 2019, gold mining destroyed 25,315 acres of land across three indigenous territories—Munduruku, Yanomami, and Kayapó—located in Brazil’s Legal Amazon (BLA). By 2020, there were over 20,000 miners reported on indigenous Yanomami reserves, mostly in Roraima state [31]. As illegal mining on indigenous lands as spread over time, there were increases in reported malaria cases among indigenous people, and in the proportion of cases reporting timber/fishing as their primary occupation, from 6.88% to 21.44% between 2016 and 2020, the primary occupation of most indigenous people. This study’s findings show that non-indigenous people, which include most miners, are more likely to report getting malaria outside Brazil, whilst indigenous people more often acquire malaria locally, with indigenous people 62% less likely to be imported cases.

Further, between 2018 and 2020, malaria cases (especially of *P. falciparum*) increased notably in young indigenous children; over the five-year period, the median age of cases among indigenous people was 12.69 years compared to 30.57 years in non-indigenous cases. This is consistent with findings a study on factors associated with malaria in indigenous populations in Amazonas state [14]. Another study in Amapá on the border with French Guiana showed that indigenous children had a disproportionate burden of malaria when compared to non-indigenous children and among indigenous populations in Venezuela [15, 32].

The increase in malaria cases among indigenous children, especially of *P. falciparum*, begs further investigation into effective approaches to optimize prevention for this population. Malaria could be transmitted to children at night while they are sleeping, so prevention measures can focus on use of long-lasting insecticidal nets (LLINs), if culturally acceptable, and house spraying when indigenous people live in villages and wood houses, whilst ensuring mining sites that contain pools of stagnant water are not in close proximity to indigenous settlements. But, as observed in SIVEP data, “children’s occupation” (which is usually their parent’s occupation) reflects that malaria could be transmitted to them while accompanying parents in activities such fishing and timber. More research is needed on delivery and sustainability of prevention measures in indigenous communities, determining when and where malaria is likely to be transmitted, especially to children.

Indigenous individuals are getting more timely treatment for both *P. falciparum* and *P. vivax* malaria, but timeliness to treatment lags for non-indigenous populations, many of whom are miners. However, gold miners and indigenous populations often live as neighbours in remote areas with less access to prompt diagnosis and treatment [33]. Thus, this finding requires further investigation as it runs counter to what is expected, given that indigenous people usually live further from health facilities and, therefore, must wait longer periods to commence treatment. The indigenous health facilities in Brazil sometimes use different malaria surveillance forms, so this finding may in part be explained by errors in classification on the forms.

In some municipalities, malaria cases are being diagnosed and notified in areas far from the municipality of infection, mainly in Boa Vista, the largest city in Roraima. Malaria cases among non-indigenous people (who are predominantly miners) in Alto Alegre municipality are often notified in Boa Vista, with the reported as place of infection as in Alto Alegre or outside Brazil, mostly in Venezuela. In contrast, malaria cases among indigenous populations in Alto Alegre are being diagnosed and reported in Alto Alegre. One hypothesis for this finding is that because gold mining in Alto Alegre is illegal and in Yanomami territory, miners may incorrectly report infections as coming from Venezuela. They also may not seek care in Alto Alegre, since most clinics there are Indigenous Health Posts. Due to the stigma associated with mining and tensions between miners and indigenous communities, miners travel back to Boa Vista or larger towns to seek treatment, do not disclose their true occupation, or do not report cases through the national health system at all, opting to purchase treatments through the informal markets.

Areas where informal mining and indigenous populations coexist are often remote, posing challenges to control and elimination efforts that require innovative solutions [34]. Both are also socioeconomic risk factors. Therefore, innovative surveillance and treatment approaches are needed to promptly identify and treat cases in these populations [26]. Prevention measures such as LLINs and diagnosis and treatment kits can be made available closer to mining sites. Additionally, careful attention must be given to the design and material of LLINs and other vector control interventions, which play a significant role in user preferences and appear to drive net use [35]. In French Guiana, Malakit targets gold miners working illegally with free malaria self-diagnosis and self-treatment kits for *P. falciparum* [36]. In light of the epidemiological shifts in malaria species composition, both prevention and treatment strategies need to use nuanced approaches to combat *P. falciparum* and *P. vivax* differently.

Along with the epidemiological shifts to greater proportions of *P. falciparum* malaria and more cases in indigenous populations, there was a shift toward younger ages. Overall, among all cases, it is concerning that there were almost six times more cases in children under five years of age in Roraima in 2020 compared to 2019. This could lead to an increased number of deaths in this age group, as there is evidence from Brazil and from other countries to show that case fatality rates are higher in younger age groups, especially for *P. falciparum* malaria [4, 37].

Malaria remains a serious threat in Roraima state, especially among high-risk populations, such as miners, migrants, and indigenous people. The transition from malaria control to elimination requires understanding of risk factors among high-risk populations in order to tailor interventions appropriately [38]. In 2016, health ministers from across the region developed a Plan of Action for Malaria Elimination 2016–2020 [39]. One of its strategic goals was “to further improve surveillance systems with early detection of cases and outbreaks and advocate collection of malaria data (by case, including information on age, sex, ethnicity, and other variables that facilitate appropriate analysis of disparities and inequalities between populations).” Malaria transmission in Roraima state is dynamic and its changing epidemiological profile should be monitored closely through robust surveillance systems and strategies tailored to specific population risk groups, considering the local context and changes in each group’s occupational tendencies and residence status [40]. Furthermore, a strong early detection and rapid response system will facilitate the detection of increased malaria cases and epidemic outbreaks at the community and sub-community levels, border regions and mining sites.

This study had several limitations. Education could not be included in the final multivariable model investigating risk factors because children’s educational status was missing or incomplete. Also, following SIVEP guidance, the occupation of children should be classified according to the category closely related to possible exposure to malaria in the last 15 days. The child’s occupation is therefore usually classified as that of their primary caregiver; however, many times the “other” category is selected, over representing this category in the analysis. A further limitation is that this is surveillance data and only data on positive cases were available, and could be incomplete. Information on negative cases would have lent to more robust analysis of risk factors.

## Conclusions

Malaria remains a serious threat in Roraima state, especially among high-risk populations, such as miners, migrants, and indigenous people. Demographic and socioeconomic profiles of malaria cases have changed over the 5-year period from 2016 to 2020, as illegal mining increased in indigenous areas and as cross-border movement decreased when the border closed to Venezuelan migrants during the COVID-19 pandemic. Though Brazil aims to eliminate *P. falciparum* malaria by 2030, it is increasing in Roraima state. Study findings show that risk profiles for imported and autochthonous malaria changed rapidly over time and were shaped by unplanned external factors such as the humanitarian crisis in Venezuela, the global COVID-19 pandemic and uncontrolled illegal mining in indigenous areas. Further, there have been delays in time from diagnosis to treatment in certain risk groups, likely because cases are notifying in areas far from the place of infection. Surveillance and intervention strategies need to be able to adapt quickly and nimbly to best match the needs and characteristics of vulnerable populations.

## Supplementary Information

**Additional file 1.**

## Data Availability

The datasets used and/or analysed during the current study are available from the corresponding author on reasonable request.

## Abbreviations

SIVEP: Malaria Epidemiological Surveillance Information System
SINAM: Notifiable Diseases Information System
SUS: National Health System
RDT: Rapid diagnostic test
LLIN: Long-lasting insecticidal nets
BLA: Brazil’s Legal Amazon
